# Ultroser G inhibits prostaglandin output by human decidual cells

**DOI:** 10.1155/S0962935192000334

**Published:** 1992


Mediators of Inflammation, 1, 213–214 (1992)

### Ultroser G inhibits prostaglandin output by human decidual cells

Dear Sir

We have recently measured prostaglandin (PG) production by freshly dispersed human term decidual cells *in vitro*.[1] To validate our protocol, we investigated the effect of a number of commercially available tissue culture media and media additives on the production of $PGF_{2\alpha}$ and $PGE_2$ by these cells.

Single cell suspensions of human term decidua were prepared by enzymic dispersion and Percoll density centrifugation as previously described.[1,2] The cells were then incubated at $2 \times 10^6$ cells/ml for 2 h in various culture media with or without media supplementation. All incubations were carried out under sterile conditions at 37°C in a humidified 5% $CO_2$/95% air environment and in the absence of antibiotics; appropriate controls were included. $PGF_{2\alpha}$ and $PGE_2$ output were measured by radioimmunoassay (RIA) of the conditioned medium using antisera raised against the PGs as their methyl oximes.[3] The tissue culture media investigated included RPMI 1640 containing Hepes buffer and 2 mM glutamine (Gibco-Europe, Uxbridge, Middlesex., UK), Minimal Essential Medium Eagle with Earle's salts (Sigma Chemical Co., Poole, Dorset, UK), Dulbecco's Modified Eagle's medium containing 1.0 g/l glucose (DMEM; Gibco-Europe) and endotoxin-free phosphate buffered saline (PBS; Sigma); medium additives included 10% heat-inactivated normal human serum (NHS; Blood Transfusion Service, John Radcliffe Hospital, Oxford, UK), 0.25% bovine serum albumin (BSA [fraction V]; Sigma) or a serum substitute, Ultroser G, added to a final concentration of 2% as recommended (Gibco-Europe).

PG output by cells cultured in RPMI, DMEM and PBS was similar. Addition of either 10% NHS or 0.25% BSA to any of these culture media had no effect on PG output; however, the addition of 2%

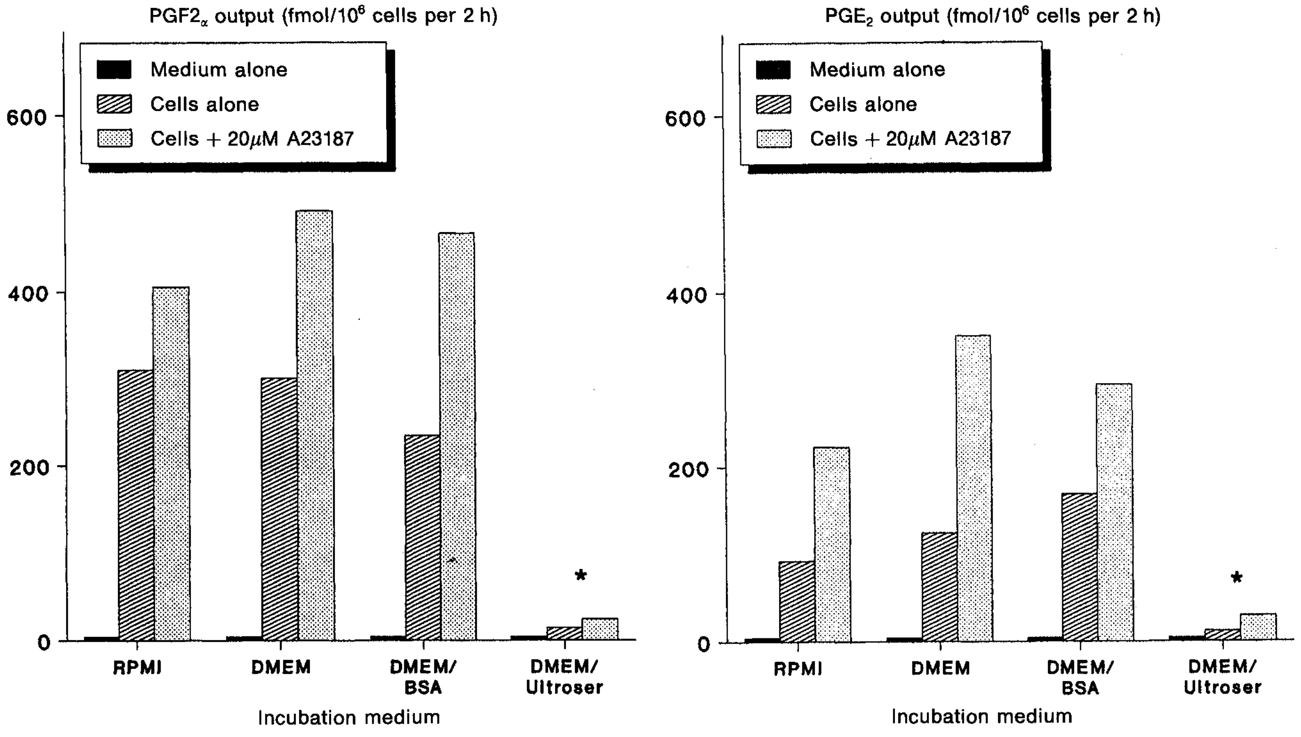

FIG. 1. Effect of Ultroser G on prostaglandin production by human decidual cells. Single cell suspensions of human term decidua were prepared by enzymic digestion and incubated in various culture media as described. $PGF_{2\alpha}$ (a) and $PGE_2$ (b) output were measured by radioimmunoassay. The presence of 2% Ultroser G (Gibco-Europe) markedly decreased measured prostaglandin levels. Values are median from three separate experiments (*$p < 0.05$ as compared with media without 2% Ultroser G).

© 1992 Rapid Communications of Oxford Ltd

Ultroser G to any of the media profoundly reduced measured levels of both $PGF_{2\alpha}$ and $PGE_2$ (Figures 1a and 1b respectively). The RIA standard curves were unaffected by the presence of 2% Ultroser G (data not shown), so it is unlikely that this medium had any affect on the performance of the assay. Moreover, 2% Ultroser G did not affect cell viability. It would seem rather that Ultroser G acts either directly or indirectly to strongly inhibit the production and/or release of PGs from term decidual cells *in vitro*, since attempts to stimulate PG production using the calcium ionophore, A23187 at a final concentration of 20 $\mu$M were equally frustrated (Figure 1).

Whether this phenomenon is specific to PGs and to our decidual cell model in particular remains unclear. We would suggest, however, that laboratories involved in prostanoid research should avoid the use of Ultroser G and that the possible mechanism of inhibition be further investigated.

*E. R. Norwitz and A. López Bernal*

*University of Oxford, Nuffield Department of Obstetrics and Gynaecology, John Radcliffe Hospital, Headington, Oxford OX3 9DU, UK*

### References

1. Norwitz ER, Starkey PM, López Bernal A, Turnbull AC. Identification by flow cytometry of the prostaglandin-producing cell populations of human term decidua. *J Endocrinol* 1991; **131**: 327–334.
2. Vince GS, Starkey PM, Jackson MC, Sargent IL, Redman CWG. Flow cytometric characterisation of cell populations in human pregnancy decidua and isolation of decidual macrophages. *J Immunol Method* 1990; **132**: 181–189.
3. Kelly RW, Deam S, Cameron MJ, Seamark RF. Measurement by radioimmunoassay of prostaglandins as their methyl oximes. *Prostaglandins, Leukotrienes Med* 1986; **24**: 1–14.

**Received 10 March 1992; accepted in final form 18 March 1992**
